# Children's tooth decay in a public health program to encourage low-income pregnant women to utilize dental care

**DOI:** 10.1186/1471-2458-10-76

**Published:** 2010-02-18

**Authors:** Peter Milgrom, Marilynn Sutherland, R Mike Shirtcliff, Sharity Ludwig, Darlene Smolen

**Affiliations:** 1 Northwest Center to Reduce Oral Health Disparities, University of Washington, Seattle, WA USA; 2 Klamath County Department of Public Health, Klamath Falls OR USA; 3 Advantage Dental Services LLC, Redmond, OR USA

## Abstract

**Background:**

A community-based public health program to provide a dental home for women covered by the Oregon Health Plan (Medicaid) in Klamath County, Oregon USA was instituted with the long-term goal to promote preventive oral care for both mothers and their new infants provided by dental managed care companies.

**Methods:**

As part of the evaluation of the program, children in Klamath and comparable non-program counties were examined in their 2^nd ^year of life to begin to determine if benefits accrued to the offspring of the mothers in Klamath County.

**Results:**

Eighty-five and 58.9% of the children were caries free in the Klamath and comparison county samples, respectively (RR = 1.48, 95% CI 1.13, 1.93). The mean (SD) number of teeth with any decay was .75 (2.5) in the test population and 1.6 (2.5) in the comparison population (t = 2.08, p = .04).

**Conclusions:**

The assessment showed that children of mothers in the Klamath County program were about one and a half times more likely to be caries free than children in the comparison counties. Additional controlled studies are being undertaken.

## Background

Healthy People 2010 established objectives to reduce disparities among preschool children in the United States (US) [1]. However, programs aimed at reducing disparities focusing solely on children may fail to identify solutions that enhance access or improve oral health. An alternative is to focus on the association between mother and child. When low-income pregnant women and mothers with infants have regular dental visits, both mother and child should experience benefits [2]. Low-income children whose mothers have a regular source of dental care are more likely to have dental visits [3] and be healthier than children of mothers who do not have a regular source of dental care [4]. Benefits are derived for the child because of anticipatory guidance [5,6] and by preventing the transmission of infectious oral bacteria from mother to child [7].

Medicaid is a federal-state program that entitles low-income pregnant women and their offspring dental treatment. States contract with for profit dental managed care companies to provide care or buy services from individual private dentists. Access is poor, largely because the number of dentists available to care for this population is inadequate.

A community-based public health program providing a dental home for women covered by the Oregon Health Plan (Medicaid) in Klamath County, Oregon USA was instituted, with the long-term goal to promote preventive oral care for both mothers and their new infants. Pregnant women received home visits or attended counseling sessions at the Women, Infant, and Children (WIC) program of the county health department, and were assigned a dental home under a dental managed care program. As a result of the program 55.8% of eligible pregnant women received care, a rate far exceeding the rate for poor women in other counties and even the state as a whole [8].

As part of the continuing assessment of the program, children in Klamath and comparable non-program counties were examined to begin to determine if benefits accrued to the offspring of the mothers in Klamath County.

## Methods

### Setting

The setting was Klamath County in rural southeast Oregon USA [8,9]. The population in 2008 was 66,425 and growth 2000 to 2008 was 4.2%. The county in 2008 was 82.7% white but not of Hispanic or Latino origin, 9.2% Hispanic, 4.1% American Indian or Alaska Native, 1.0% Asian, 0.8% black, 0.2% Pacific Islander and 2% other or mixed races. There was no artificial fluoridation and little naturally occurring fluoride. Over half (432, 51.6%) of the 836 births in Klamath County in 2007 were to low-income women covered by the Oregon Health Plan (OHP), the Medicaid program.

### Participants

Two groups of children were examined. The first group consisted of a sample of offspring of mothers in Klamath County who were eligible for the counseling program and received dental care during pregnancy or within two months of delivery. The children of 235 mothers were invited for examinations in random order and 113 (48%) were recalled and examined. The primary reason for non-participation was that the program was unable to contact the mother after multiple attempts. The comparison group consisted of 56 children in Deschutes, Crook and Jefferson counties. The children were selected from a list of all children between 24 and 35 months of age enrolled with OHP and eligible for care by the dental care organization Advantage Dental Services, LLC. The universe of eligible children was identified and the parents contacted by telephone. In Deschutes County there were 70 children identified: 30 (42.8%) were examined. In Jefferson County there were 37 children eligible: 16 (43.2%) participated. In Crook County there were 22 eligible children: 10 (45.4%) participated. All three counties are in rural Oregon and have similar levels of births to women served by the Oregon Health Plan. The Institutional Review Board of the University of Washington approved the study and the informed consent of the parents was obtained.

### Examinations

The examinations were conducted by one of the dental managed care organizations in cooperation with the Klamath County Department of Health as part of the ongoing evaluation of the program. Visual examinations were conducted using artificial light and a front plane mirror by two examiners (Shirtcliff and Woll). World Health Organization criteria [10] were used: only frank cavitation was recorded as tooth decay. Dental exams were performed in dental offices in Bend, (Deschutes County) Madras (Jefferson County) and Prinville (Crook County). The detection of caries was done using visual/tactile examination, using a #23 explorer without significant axial force. The interrater reliability of the examiners was assessed on seven children seen by both examiners: the intraclass correlation (ICC) for the number of teeth with decay was .95. Examiners were not blind to which sample was being examined. The outcome measures in the study were the number of children with any decayed deciduous tooth and the number of such teeth.

### Descriptive information on the children and their mothers

The child's age, gender and race, and whether this was the mother's first child were collected by interview or from the WIC database. For the comparison group, whether the mother received any dental care during her pregnancy or immediately post partum was obtained from the dental managed care organizations.

### Analysis plan

The de-identified data were recorded in an Excel spreadsheet by program personnel, edited, and then imported into SPSS (version 16 for Mac). We tested the hypothesis that children of mothers in the Klamath County program would be less likely to have any tooth decay and have fewer decayed or filled teeth than the children of mothers in the comparison counties. Binomial regression using a log link was used to calculate the relative risk of being caries free, adjusted for child age and Hispanic race. The binomial regression was implemented using generalized estimating equations with a robust variance estimator to estimate valid standard errors and perform statistical inference [11].

## Results

The average ages of the children in the program evaluation were 24 and 28 months for Klamath County and the comparison group, respectively (t = 3.0, p < .003). The Klamath children were 50% male while the comparison county children were 55 percent male (p > .05). Twenty-eight of 50 children (56%) in the comparison group were Hispanic while a smaller proportion of the children (15/92, 16%) examined from the Klamath County were Hispanic (Fisher's Exact Test, p = .001). This was the first child for 47.3 percent (44/93) mothers in the Klamath population and 47.2 percent (25/53) in the comparison population.

All of the mothers of children examined in Klamath had received dental care; about half of the women in the comparison group received dental care during pregnancy or in the two-month window post partum covered by the Oregon Health Plan. The high rate of care during pregnancy in the comparison county reflects the ongoing efforts of the state health department and the dental care organizations to promote dental care for low-income pregnant women throughout the state.

**Figure 1:**
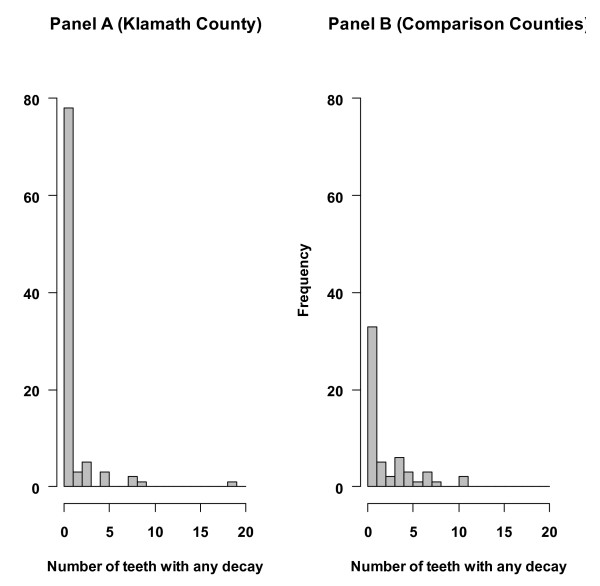
**Distribution of the number of teeth/child with any decay by group**.

Eighty-five percent (96/113) and 58.9 percent (33/56) of the children were caries free in the Klamath and comparison county samples, respectively (Fisher's Exact Test, p < .0004). The mean (SD) number of teeth with any decay was .75 (2.5) in the Klamath population and 1.60 (2.5) in the comparison population (t = 2.08, p = .04). Figure 1 gives the distribution. The relative risk for being caries-free (Klamath County versus comparison counties), adjusted for child age and Hispanic race, is 1.48 (95% Confidence Interval 1.13, 1.93).

## Discussion

Previously we have shown that a counseling program based in the WIC program of a county health department increased the utilization of dental care during pregnancy among low-income mothers in rural Oregon [9]. This program included educational materials intended to promote dental visits for the offspring in the second year of life. The preliminary findings from this outcomes assessment are consistent with previous literature and suggest this intervention during pregnancy likely had benefits for the child. In previous work we have shown that low-income mothers who rate their own oral health as better are more likely to take their child to the dentist [12]. Other work has shown that the majority of children will see the same dentist as their mothers [3]. This will be especially true in rural areas when there are almost no pediatric specialists.

The outcomes assessment was conducted by the public health department as part of its ongoing responsibility to the community and was not a rigorous experiment. Nonetheless it was based on sound principles that were derived from controlled trials and other studies. Participants could not be assigned to conditions randomly and the comparison population is not identical to the program county. Thus, statistical methods were used to adjust for differences in the populations that are a threat to the validity of the conclusions. The number of children examined was relatively small and examiners were not blinded to treatment condition. We do not know anything about the treatments actually provided by dentists. Thus, conclusions drawn from this preliminary work should be conservative. A randomized clinical trial involving four additional counties in Oregon is now being conducted and should allow more definitive conclusions to be drawn.

Addressing the growing rate of early childhood caries in US children from low-income families is vexing and seemingly impossible without changing the paradigm or treatment model that underlies the approach. The predominant approach in the US, embodied in the Medicaid program, is to focus on the child alone. Benefits for mothers are much more limited, and practically speaking; dental care is inaccessible because of the unwillingness of states to provide coverage and the low fees in Medicaid adult programs. Workforce shortages further exacerbate the problem.

In spite of the efforts described here there remain barriers to dental care for pregnant women. A recent survey of general practitioners in Oregon found that while attitudes toward care of pregnant women were generally positive, some dentists held incorrect beliefs about aspects of care and others were afraid of being sued in the event of poor pregnancy outcomes [13]. Collaborative efforts are underway with the managed care plans and the State of Oregon to distribute correct information to dental offices and meetings are being held with obstetric care providers to enhance inter-professional communication. Similar efforts will be needed to address the reluctance of dentists to see very young children.

## Competing interests

The authors declare that they have no competing interests.

## Authors' contributions

PM helped conceive of the public health program, analyzed the data, and wrote the manuscript. MS directed the public health program and contributed to the manuscript. RMS was responsible for the clinical examinations and contributed to the manuscript. LS compiled the program data and provided the demographic information for the manuscript. DS supervised data collection, worked with community partners and contributed to the manuscript. All of the authors have read and approved the final manuscript.

## Pre-publication history

The pre-publication history for this paper can be accessed here:

http://www.biomedcentral.com/1471-2458/10/76/prepub
